# miR-21 and Pellino-1 Expression Profiling in Autoimmune Premature Ovarian Insufficiency

**DOI:** 10.1155/2020/3582648

**Published:** 2020-04-13

**Authors:** Xinran Li, Jiaxin Xie, Qingru Wang, Huihua Cai, Chuhai Xie, Xiafei Fu

**Affiliations:** ^1^Department of Obstetrics and Gynecology, Zhujiang Hospital of Southern Medical University, Guangzhou, Guangdong, China; ^2^Department of Obstetrics and Gynecology, Guangdong Provincial People's Hospital, Guangdong Academy of Medical Sciences, Guangzhou, Guangdong, China; ^3^Department of Orthopedics, The Second Affiliated Hospital of Guangzhou Medical University, Guangzhou, Guangdong, China

## Abstract

**Background:**

Premature ovarian insufficiency (POI) represents the hypergonadotropic hypoestrogenic symptoms that result in the loss of ovarian follicles. 5-30% POI cases are suggested to be involved in autoimmune etiology. MicroRNA-21 (miR-21) plays a vital role in ovarian folliculogenesis via regulating and interacting with multiple target genes. Here, we conduct the target prediction of miR-21, identify the expression and correlation of miR-21 and its putative target Pellino-1 (Peli1), and confirm their relationship with clinical characteristics in autoimmune POI.

**Methods:**

Bioinformatic analysis was conducted to screen the miR-21 putative target gene. Autoimmune POI mouse models were established by ZP3 immunization. Serum miR-21, Peli1 mRNA of peripheral blood mononuclear cells (PBMCs) and regulatory T cells (Tregs), general status, spleen Tregs ratio, inflammatory factors, ovarian endocrine function, and ovarian structure were evaluated. For autoimmune POI patients, serum miR-21, PBMCs Peli1 mRNA levels, general data, immune parameters, hormone levels, and ultrasound examinations were obtained. The correlations of miR-21 with Peli1 and clinical characteristics in patients were analyzed.

**Results:**

Peli1 was selected based on four microRNA prediction databases and literature retrieval. In mouse models, serum miR-21 level, PBMCs and Tregs Peli1 mRNA, and spleen Tregs ratio were 0.61 ± 0.09, 0.12 ± 0.12, 0.27±0.23 and 4.82 ± 0.58, respectively, lower than those in the control group. In patients, miR-21 level (0.60 ± 0.14) and Peli1 mRNA (0.30 ± 0.14) were lower than those in the control group (1.01 ± 0.07 and 1.63 ± 0.54); miR-21 was positively related with Peli1, AMH, E_2_, the size of the uterus, and ovarian volume and negatively related with FSH, LH, and the number of positive immune parameters (AOAb, EMAb, ACL, ANA, ds-DNA, ACA, IgG, IgA, IgM, IgE, C3, and C4).

**Conclusions:**

Low expressions of miR-21 and Peli1 were detected in autoimmune POI mice and patients. Positive correlation between miR-21 and Peli1 was observed in autoimmune POI patients, suggesting that miR-21 and Peli1 might be associated with the pathogenesis of autoimmune POI.

## 1. Introduction

Premature ovarian insufficiency (POI), which occurs in women before the age of 40, is defined as primary or secondary amenorrhea for more than 4 months and increased follicle-stimulating hormone (FSH) greater than 25 mIU/mL on two occasions [1]. Multiple etiologies have been reported to be associated with POI, including genetic, iatrogenic, autoimmune, metabolic, and environmental factors [2, 3]. About 5-30% of clinically evident POI has been previously diagnosed with autoimmune diseases [4, 5]. Previous studies suggested that 30-40% POI cases were mostly concomitant with thyroid diseases [6], then followed by Addison's disease, rheumatoid arthritis (RA), systemic lupus erythematosus (SLE), vitiligo, idiopathic thrombocytopaenic purpura (ITP), diabetes mellitus (DM), etc. [7–9]. Currently, the mechanisms of autoimmune POI still remain poorly understood. Therefore, specific guidelines and effective treatments are not available.

MicroRNAs (miRNAs) are 22–24 nt endogenous and noncoding small RNAs [10]. Among them, microRNA-21 (miR-21) plays an essential but incompletely understood role in the reproductive system. Transcriptomic data analyses suggested that miR-21 expressed in zebrafish primary growth and previtellogenic follicles, which might mediate posttranscriptional control in follicle activation [11]. miR-21 is also expressed in sheep follicles and became a potentially important regulator of the follicular-luteal transition [12]. Additionally, miR-21 was found to be involved in the regulation of cell survival, steroidogenesis, and differentiation during follicle selection and ovulation in the monovular equine ovary [13]. Similarly, our previous studies demonstrated that the overexpression of miR-21 is associated with the alleviation of granulosa cell (GC) apoptosis and partially recovered the chemotherapy-induced ovarian damage, which was characterized by increased follicle counts, E_2_ level, and ovarian weights and decreased FSH levels [14]. However, the correlation between miR-21 and autoimmune POI has not been elucidated.

Generally, a single miRNA can interact with multiple targets and vice versa [15]. Pellino-1 (Peli1), a critical E3 polyubiquitin ligase in immune tolerance, is one of the putative miR-21 targets. Marquez et al. [16] revealed that the overexpression of miR-21 inhibited a Peli1 3′-UTR luciferase reporter in cultured liver cells. Yuan et al. [17] demonstrated that low Peli1 and high miR-21-5p levels were observed in classical Hodgkin lymphoma (cHL) cell lines. In addition, Luther et al. [18] showed that mesenchymal stem cell exosomes upregulated miR-21a-5p levels, thereby downregulating the expression of the proapoptotic genes Peli1, PDCD4, and PTEN in the myocardium. Peli1 has been reported to be abundantly expressed in T lymphocytes and functions as a negative regulator in T cell activation. It is associated with multiple autoimmune diseases such as systemic lupus erythematosus (SLE), multiple sclerosis (MS), and autoimmune encephalomyelitis (EAE) [19–21]. The overexpression of Peli1 led to improved self-tolerance and immunosuppression. Conversely, Peli1-deficient mice were susceptible to spontaneous autoimmune disease, which indicated the loss of self-tolerance [22]. Therefore, we hypothesized that miR-21 might be involved in the development of autoimmune POI by downregulating Peli1.

In this study, we predicted the putative target genes of miR-21 and focused on Peli1. Then, we detected the expressions of miR-21 and Peli1 in autoimmune POI patients and mouse models. Finally, we explored the correlation between miR-21 and Peli1 in patients to investigate how these parameters are associated with clinical characteristics including AMH, E_2_, FSH, LH, the size of the uterus, ovarian volume, and the number of positive immune parameters (AOAb, EMAb, ACL, ANA, ds-DNA, ACA, IgG, IgA, IgM, IgE, C3, and C4). This study represents the first attempt to evaluate the relationship between miR-21 and Peli1 expressions and assert their importance in autoimmune POI.

## 2. Methods

### 2.1. Animals

Female BALB/c mice (7-8 wks; 18-22 g) were obtained from the laboratory animal center of Southern Medical University. All rats had unrestricted access to food and water under controlled temperature (30 ± 2°C) and light conditions (14 h light, 10 h dark). Vaginal smear of each mouse was obtained at 8:00-9:00 every morning. Mice with normal estrous cycle were included in this experiment.

Peripheral blood was obtained from the eyeballs of all 24 mice. For serum miR-21 and hormone examination, the whole peripheral blood of 6 mice in each group was immediately centrifuged at 3000 rpm for 10 min at 4°C to isolate the serum. The serum of each mouse was frozen in 1 mL vials and was stored at −20°C for further examination. To acquire PBMCs, the red blood cells (RBC) were removed from the rest of the blood specimens using RBC lysing (Cedarlane, Ontario, Canada) following the standard protocol. Samples were then centrifuged at 12000 rpm, for 10 min at room temperature. Then, the supernatant was discarded, and the mononuclear cell precipitation was washed twice with phosphate-buffered saline (PBS). The viability and concentration of isolated PBMCs were traditionally measured by manual counting with trypan blue (TB) using a hemacytometer.

The spleen and bilateral ovaries of all 24 mice were exteriorized for further examination. All research conformed to animal protocol in compliance with the Guidelines for the Care and Use of Laboratory Animals published by the National Institutes of Health. The protocol was approved by the Committee on the Ethics of Animal Experiments of Southern Medical University (grant number 2018A030313167).

### 2.2. Establishment of Autoimmune POI Mouse Model

A total of 24 mice with normal estrous cycle were randomly assigned to the autoimmune POI and control groups (12 mice each group). Murine ZP3 330–342 peptides (NSSSSQFQIHGPR, purity ≥ 90%) (Jetway Biotech Co., Ltd., Guangzhou, China) were dissolved in threefold-distilled water at 1 mg/mL and sterilized through ultrafiltration. Then, ZP3 solution was emulsified in an equal volume of complete immune adjuvant (CFA) or incomplete immune adjuvant (IFA). Mice of the autoimmune POI group received the first immunization of subcutaneous injection in the hind footpad and abdomen with 0.15 mL CFA emulsion containing 75 *μ*g ZP3 peptides. After 14 days since the first immunization, the mice received the secondary immunization of subcutaneous injection in the same sites with 0.15 mL IFA emulsion containing 75 *μ*g ZP3 peptides. Meanwhile, the same volume of physiological saline was injected into the same sites of mice in the control group. Our preexperiment showed that most of the mice in the autoimmune POI group presented irregular or prolonged estrous cycle after 6 weeks since the second immunization, which revealed that the mouse models were successfully established. Hence, the mice of the two groups were sacrificed at 6 weeks after the second immunization.

### 2.3. Patients and Study Design

This trial was conducted in Zhujiang Hospital of Southern Medical University according to Helsinki Declaration. Institutional review board approved the study, and written informed consent was obtained from all patients prior to participating in the study (permit number: 2019SL0027).

A total of 56 women were enrolled in this study. Women with initial diagnosis of autoimmune POI were eligible to participate in the autoimmune POI group (*n* = 26). Autoimmune POI was characterized by the age of onset before 40 years old, FSH levels greater than 25 mIU/mL on 2 occasions (≥4 weeks), and accompanied with autoimmune disorders and/or autoimmune antibodies. The control group (*n* = 30) was constituted by women aged 18-40 years old, who had regular menstrual cycle, normal hormone levels (AMH, E_2_, FSH, and LH) in the third day of menstrual period, and negative immune parameters.

In both the autoimmune POI and control groups, women who received hormone and/or immunosuppressive therapy (≤3 months), oophorectomy, pelvic irradiation, and prior chemotherapy treatment were excluded from the study. Moreover, women who suffered from thromboembolic processes, severe hypertension, severe obesity, hepatic or renal insufficiency, ovarian abnormalities, and known karyotype abnormalities were also excluded.

The peripheral blood was collected from each patient. For the autoimmune POI group, the blood was obtained at the first diagnosis of autoimmune POI. For the control group, the blood was preferentially obtained at the third day of menstrual period. Examination of hormone levels and immune parameters was finished by the Clinical Laboratory of Zhujiang Hospital of Southern Medical University. Serum and PBMCs were obtained from the whole blood specimens as previously described.

### 2.4. General Data

General data of patients in the two groups were collected at the beginning of the study, including age, BMI, menopause symptoms, age of menarche, menstrual period, menstrual cycle, gravidity, and parity.

### 2.5. Estrous Cycle

Estrous cycles of mice were detected by daily examination of vaginal smears. Briefly, vaginal secretions were collected with fine tip pipets, then dyed with methylene blue and observed by phase-contrast microscopy.

### 2.6. Ovarian Structure and Follicle Counts

The monolateral ovary of each mouse was fixed in 4% paraformaldehyde and embedded in paraffin. Ovaries were then sectioned into 5 *μ*m thickness and stained with hematoxylin and eosin (HE). The HE-staining slides were scored in a blinded manner to evaluate the degrees of autoimmune oophoritis under light microscopy. According to the previous studies, autoimmune oophoritis is characterized by lymphoplasmacytic infiltration and/or ovarian interstitial fibrosis with ovarian atrophy and/or loss of ovarian follicles. For follicle counts, the follicles were further classified into four phenotypes based on the morphologic characteristics: primordial follicles had a single layer of squamous granulosa cells, primary follicles contained at least three cuboidal granulosa cells in a single layer, secondary follicles had at least two layers of granulosa cells without an antral space, and antral follicles had at least two layers of granulosa cells with an antral space.

### 2.7. The Ratio of Spleen Tregs in Mice

The spleens were disrupted in Hanks' balanced salt solution containing 2% fetal bovine serum (FBS), and the aggregates and debris were removed through a 70 *μ*m mesh. Then, splenocytes were directly disposed with RBC lysis buffer, then washed twice with PBS and resuspended. A total of 2 × 10^6^ cells were conjugated with the following antibodies and respective isotype controls (eBioscience, Inc., CA, USA): anti-mouse CD4 (FITC), anti-mouse CD25 (APC), Rat IgG2a isotype control (PE) for 30 min, and then the cells were fixed and permeabilized with the FIX & PERM Cell Permeabilization Kit (Life Technologies, USA) according to the manufacturer's protocol. Subsequently, the cells were blocked by Fc and then stained with anti-mouse/Rat Foxp3 (PE). Finally, the cells were washed twice and resuspended in a staining buffer prior to flow cytometry analysis.

### 2.8. Isolation of Tregs in Mice

Splenocyte suspension was centrifuged at 1000 rpm for 10 min, and the remaining cells were resuspended in PBS containing 2% FBS and 1 mM EDTA. CD4+CD25+ cells were isolated from splenocyte single-cell suspensions using the EasySep™ Mouse CD4+CD25+ Regulatory T Cell Isolation Kit II (STEMCELL Technologies, Vancouver, Canada) according to the manufacturer's protocols. First, CD4+ T cells were acquired using EasySep™ Mouse CD4+ T Cell Isolation Cocktail (19852C.1). Then, the CD25+ T cells were selected using EasySep™ Mouse CD25 Regulatory T Cell Positive Selection Cocktail (18782C). Finally, the CD4+CD25+ Tregs were separated without columns using an EasySep™ magnet.

### 2.9. Quantitative Real-Time PCR (qRT-PCR)

The expression of miR-21 and Peli1 of both mice and patients was detected using quantitative real-time PCR. The total RNA was extracted from isolated PBMCs, CD4+CD25+ Tregs, and serum of mice or patients' samples using TRIzol (Invitrogen, USA). cDNA was synthesized from 2 *μ*g of the total RNA using the cDNA synthesis kit (Promega, Madison, USA). Primers were synthesized by Yeshan Biology Co., Ltd. (Guangzhou, China). Quantitative real-time PCR was performed using specific primers on the ABI PRISM® 7500 Sequence Detection System. Relative expression levels were calculated by the formula 2^−*ΔΔ*CT^. The primers used in this study are provided in Table 1.

### 2.10. ELISA

Levels of serum anti-Müllerian hormone (AMH) and ovary inflammatory factors of mice were examined using the ELISA assay. The AMH was detected by the Ultrasensitive AMH/MIS ELISA kit (Roche Co., Ltd., Switzerland). The other side of the ovary from mice was made into a homogenate under sterile conditions for the examination of IFN-*γ* and IL-10. The ovarian homogenate was quantified via the ELISA kit (Life Technologies Co., NY, USA). All procedures were performed as instructed by the manufacturer's protocol.

### 2.11. Radioimmunoassay (RIA)

Radioimmunoassay was used for the detection of the serum follicle-stimulating hormone (FSH) of mice. The examination was done by the Beijing North Institute of Biological Technology Co., Ltd., Beijing, China.

### 2.12. Chemiluminescence

The level of estradiol (E_2_) of mice was measured using a serum by an automated chemiluminescence system (Bayer Co., Ltd., Leverkusen, Germany). All procedures were performed following the manufacturer's protocol.

### 2.13. Ultrasound Examination

Ultrasound examination was conducted at the same day of blood collection to evaluate the size of the uterus, ovarian volume, and endometrial thickness. Scans were obtained by a single observer using the Acuson 128 XP10 model (Mountain View, CA). Longitudinal and transverse views of the uterus were obtained, and uterine length (from the top of the fundus to the cervix), anteroposterior and transverse diameters of the fundus, and endometrial thickness (the maximum endometrial diameter between the echogenic interfaces of the myometrium and endometrium) were measured. The uterus volume was calculated using the formula for a modified prolate ellipsoid (length × anteroposterior diameter × transverse diameter/2). Longitudinal and transverse views of the ovaries were obtained for the measurement of length, breadth, and depth of each ovary. Ovarian volume (right and left) was calculated (depth × breadth × length/2). Average volumes of the right and left ovaries of each patient were used for comparisons.

### 2.14. Bioinformatics and Analysis

Target genes of miR-21 were predicted by miRDIP (http://ophid.utoronto.ca/miRDIP/index.jsp), TargetScan5.1 (http://www.targetscan.org/), miRecords (http://c1.accurascience.com/miRecords/), and miRanda (http://www.microrna.org/). Genes common among the 4 databases were further selected after literature consultation.

### 2.15. Statistical Analysis

All statistics were analyzed by SPSS 22.0. Continuous variables are summed up by their mean ± SD. Independent sample *t* tests were used to compare baseline characteristics between the control and autoimmune POI groups when the variables were normally distributed, and the Mann–Whitney *U* test was used in cases of nonparametric distribution. Pearson's exact *χ*^2^ and Fisher's exact tests were used for enumeration data. Spearman's correlation analysis was performed to evaluate the correlations of miR-21 and Peli1 with related factors. All tests were considered significant when *P* < 0.05.

## 3. Results

### 3.1. Animal Experiments

#### 3.1.1. General Status

Six weeks after the second immunization, the mice of the autoimmune POI group displayed modest growth retardation and appeared with depression-like behavior including dull movement, weakened glossy hair, reduced food intake, staggering gait, and weight loss. Moreover, smaller uterus and lower ovarian volume were observed in the autoimmune POI group compared to the control group.

#### 3.1.2. AMH, E_2_, and FSH Levels

**Figure 1:**
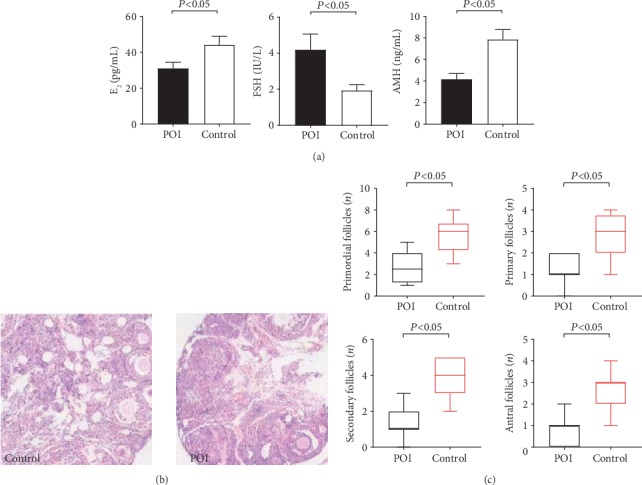
Ovary function and structure of mice. (a) The levels of E_2_ and AMH decreased while the level of FSH significantly increased in the autoimmune POI group. (b) Ovarian structure was observed under light microscopy (×40) after embedding and staining. Follicles of every stage were legible in the control group. And the amounts of follicles decreased in autoimmune POI mice. (c) Follicle counts were observed under light microscopy. The amounts of follicles of every stage obviously are lower in the autoimmune POI group.

The serum levels of E_2_(30.97 ± 3.51 pg/mL) and AMH (4.17 ± 0.52 ng/mL) in the autoimmune POI mice were significantly lower than those in the control group (44.09 ± 4.87 pg/mL, 7.87 ± 0.91 ng/mL), while the serum levels of FSH (4.20 ± 0.86 IU/L) significantly increased (*P* = 0.001) (Figure 1(a) and Table 2).

### 3.2. Ovarian Structure and Follicle Counts

The ovarian morphology in the control group was presented in red color and white punctiform prominences on the surface, whereas the autoimmune POI group was presented in pale white with fewer white punctiform prominences. The microscopic examination revealed no inflammation in the mice of the control group. Conversely, severe lymphoplasmacytic inflammatory infiltration was observed in the developing follicles of the autoimmune POI group (Figure 1(b)). However, the amounts of follicles at different stages in the autoimmune POI group were significantly lower than those in the control group (*P* < 0.05) (Figure 1(c)).

### 3.3. Inflammatory Factors

**Figure 2:**
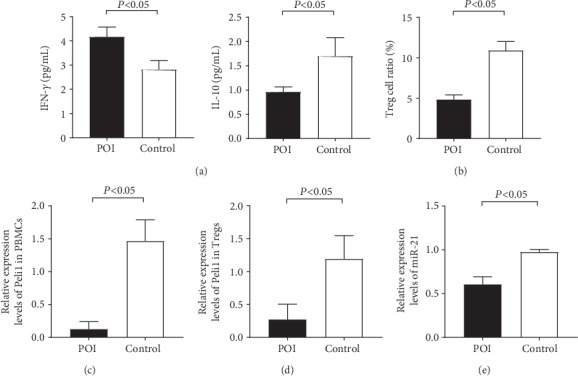
Immunologic function and Peli1 expression in autoimmune POI mouse models. (a) The levels of inflammatory factors were tested using the ovary homogenate by the ELISA assay. In the autoimmune POI group, the expression of IFN-*γ* increased while IL-10 decreased. (b) Flow cytometry analysis was performed for Treg cell sorting. The ratio of Treg cells in the autoimmune POI group was apparently lower. (c–e) qRT-PCR was performed to detect the relative expressions of Peli1 mRNA and miR-21. Peli1 mRNA quantitation in both PBMCs (c) and spleen Treg cells (d) and serum miR-21 (e) were significantly lower in the autoimmune POI group.

Compared with the control group, the mice in the autoimmune POI group had higher IFN-*γ* (4.17 ± 0.40 pg/mL) (*P* ≤ 0.001) and lower IL-10 (0.96 ± 0.10 pg/mL) (*P* ≤ 0.001) levels (Figure 2(a)).

### 3.4. The Ratio of Tregs in the Spleen

The Tregs ratio in the spleen of the autoimmune POI group (4.82 ± 0.58%) significantly decreased compared with that of the control group (11.07 ± 1.41%) (*P* ≤ 0.001) (Figure 2(b)).

### 3.5. Peli1 mRNA and miR-21 Expressions

In PBMCs, the level of Peli1 in the autoimmune POI group (0.12 ± 0.12) was significantly lower than that in the control group (1.47 ± 0.32) (*P* ≤ 0.001) (Figure 2(c)). Meanwhile, in spleen Tregs, the level of Peli1 in the autoimmune POI group was 0.27 ± 0.23, also lower than that in the control group (1.19 ± 0.35) (*P* ≤ 0.001) (Figure 2(d)). Similarly, the expression of miR-21 was apparently lower in the autoimmune POI mice (0.61 ± 0.09) (*P* ≤ 0.001) (Figure 2(e)).

### 3.6. Bioinformatics Analysis for miR-21

**Figure 3:**
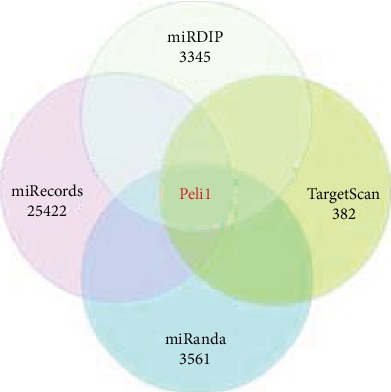
miR-21 target genes in four databases.

Genes targeted by miR-21 were screened using four prediction databases, including miRDIP, TargetScan, miRecords, and miRanda. Peli1 was chosen as a target for further research (244 common genes in four databases) (Figure 3).

## 4. Clinical Trial Sample

### 4.1. General Data

Demographic characteristics of the participants are shown in Table 3. There was no significant difference in age and BMI between the two groups. Notably, autoimmune POI patients manifested the earlier age of menarche than the control group (*P* = 0.016). Moreover, shortened menstrual period (*P* ≤ 0.001), prolonged menstrual cycle (*P* ≤ 0.001), decreased gravidity (*P* ≤ 0.001), and reduced parity (*P* ≤ 0.001) in the autoimmune POI group were also observed (Table 3).

### 4.2. Hormone Levels

As expected, patients in the autoimmune POI group had higher mean FSH value (78.27 ± 40.39 IU/L) and LH value (38.78 ± 22.31 IU/L) than patients in the control group (FSH = 7.57 ± 2.72 IU/L, *P* ≤ 0.001; LH = 6.12 ± 6.42 IU/L, *P* ≤ 0.001). On the contrary, the mean AMH values (0.05 ± 0.08 *μ*g/L) and E_2_ values (117.60 ± 131.15 pmol/L) were significantly lower than those of the control group (AMH = 1.73 ± 0.26 *μ*g/L, *P* ≤ 0.001; E_2_ = 268.70 ± 279.03 pmol/L, *P* ≤ 0.001). However, the mean free triiodothyronine (fT3), free thyroxin (fT4), and thyroid-stimulating hormone (TSH) values in the autoimmune POI group had no significance compared to those in the control group (Table 4).

### 4.3. Ultrasound Examination

**Figure 4:**
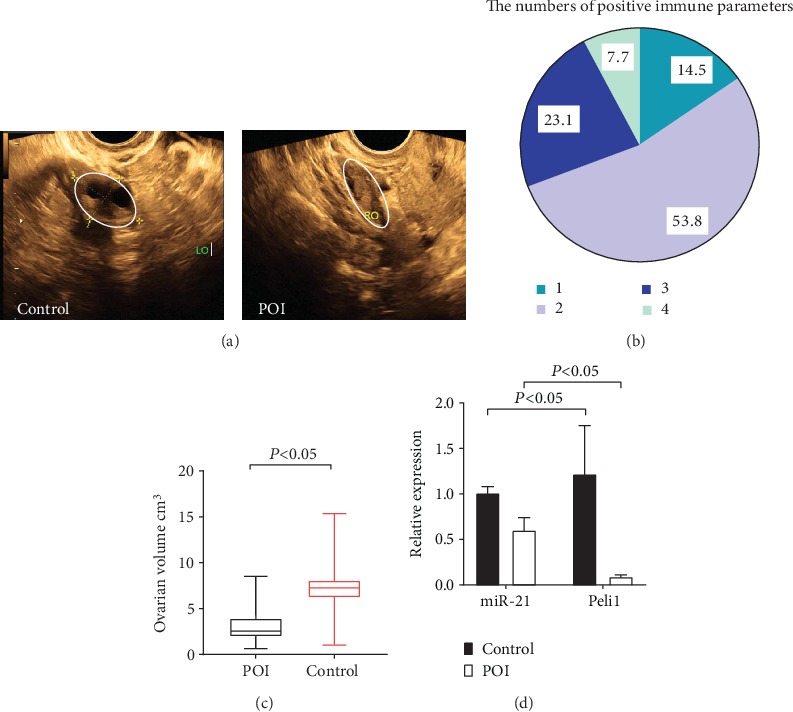
Ovary structure and immune parameters of patients. (a) Antral follicles are observed in the ovary of a normal-cycling woman while no follicle images are seen in an autoimmune POI patient under ultrasonography. (b) Antibodies and immunity factors were considered immune parameters. Two or more positive immune parameters were found in 85.5% of autoimmune POI patients. (c) Ovarian volume was measured with ultrasound examination and decreased in the autoimmune POI group. (d) Peli1 mRNA was measured by qRT-PCR in PBMCs isolated from the peripheral blood of each participant of both groups. The relative expression of Peli1 mRNA in autoimmune POI patients is dramatically lower than normal-cycling women.

Antral follicles were observed in the ovaries of the control group while no follicle images were seen in the autoimmune POI group under ultrasonography (Figure 4(a)). The size of the uterus and ovarian volume in the autoimmune POI group were 25.12 ± 15.75 cm^3^ and 3.05 ± 1.87 cm^3^, respectively, lower than those in the control group (42.54 ± 14.25 cm^3^, *P* ≤ 0.001 and 7.20 ± 2.28 cm^3^, *P* ≤ 0.001) (Figure 4(c)). There were significant differences between the two groups in the size of the uterus and ovarian volume, but not in endometrial thickness.

### 4.4. Immune Parameters

In the present study, serum antibodies and immunity factors were collectively referred to as immune parameters. Serum antibodies include anti-ovarian tissue antibody (AOAb), anti-endometrial antibody (EMAb), anti-cardiolipin antibody (ACL), anti-nuclear antibody (ANA), anti-double-stranded DNA antibody (ds-DNA), and anti-adrenal cortical antibody (ACA). In addition, related immunity factors contain IgG, IgA, IgM, IgE, and serum complements 3 and 4 (C3 and C4). In both groups, the number of positive antibodies and related immune factors were summed up to calculate the number of positive immune parameters (Figure 4(b)).

### 4.5. Peli1 mRNA and miR-21 Expressions

miR-21 and Peli1 mRNA levels in the autoimmune POI group (0.60 ± 0.14 and 0.30 ± 0.14) were significantly lower than those of the control group (1.01 ± 0.07, *P* ≤ 0.001 and 1.63 ± 0.54, *P* ≤ 0.001) (Figure 4(d)). And a positive correlation of Peli1 with miR-21 was observed (*r* = 0.719, *P* ≤ 0.001).

### 4.6. The Correlations of miR-21 and Peli1 with Related Factors in Autoimmune POI Patients

There were trends demonstrating that miR-21 and Peli1 were both positively related to AMH, E_2_, size of the uterus, and ovarian volume, while negatively related to FSH, LH, and the number of positive immune parameters (Tables 5 and 6).

## 5. Discussion

Clinically, autoimmune POI has no clear and unified diagnostic criteria. Exploring the pathogenesis of autoimmune POI is vital for its diagnosis and treatment. Previous studies have suggested that the presence of miRNAs in the ovaries elucidate their potential role in regulating diverse biological processes underlying the ovarian functionality. Among them, miR-21, the earliest discovered miRNAs in various carcinomas, has been found to be involved in ovarian reserve. Mase et al. [23] performed a high-throughput sequencing of EIF2C2-bound miRNAs, finding that miR-21 played a functional role in 3 human granulosa-derived cell lines and primary human GCs. Karakaya et al. [24] conducted a miRNA microarray in cumulus cells, and the elevated expressions of miR-21-5p/-3p were observed. Additionally, Donadeu et al. [25] used qRT-PCR to confirm the involvement of highly expressed miR-21-5p/-3p in granulosa and theca cells during follicle atresia. In our study, we showed the lower expression of miR-21 in both autoimmune POI patients and mouse models compared with that in the control subjects. Next, in patients, we confirmed the negative correlations of miR-21 with FSH and LH and verified the positive correlations of miR-21 with AMH and E_2_, the size of the uterus, ovarian volume, and the number of positive immune parameters. This information is consistent with previous studies, which will give insights into the role of miR-21 in autoimmune POI.

miR-21 plays an essential role in the cellular and physiological pathways in the reproductive system via regulating target genes. Tian et al. [26] found that elevated miR-21 might maintain the porcine estrous cycle via targeting genes that are related to the mTOR, apoptosis, and steroidogenesis pathways. Donadeu et al. [25] confirmed that miR-21-5p/-3p were expressed at a higher level in atretic follicles, and their target genes HIF1A, ETS1, and MSH2 were downregulated during follicle atresia. Additionally, Pan and Li [27] conducted luciferase gene reporter assays and presented a binding site of miR-21 in the 3′-UTR of TIMP3 mRNA, at which miR-21 can downregulate TIMP3 to promote cumulus expansion and oocyte maturation. In the present study, a bioinformatics analysis was performed to screen the potential targeting genes of miR-21 based on four prediction databases. Among the genes common to all four databases, we selected Peli1, a member of the RING finger E3 ubiquitin ligase family, as a target for further research because of its key role in various immune or inflammatory regulation pathways [28–30]. Liu et al. [21] and Chang et al. [22] reported that Peli1-deficient mice spontaneously developed SLE that was characterized by a severe lupus-like damage and autoantibody production. On the contrary, a previous study proposed that Peli1-deficient mice resulted in reduced viral loads in tissues and attenuated brain inflammation in EAE [29]. Another study conducted by Luo et al. [20] also found that upon intranasal infection with vascular stomatitis virus (VSV), the Peli1-deficient mice displayed with reduced brain viral titer and increased survival rate in the central nervous system (CNS) [19]. However, whether Peli1 is associated with the development of autoimmune POI has yet to be explored.

The present study demonstrated that in autoimmune POI patients, the expression of Peli1 mRNA in PBMCs was lower than those in the control group. Additionally, in mouse models, a similar trend in PBMCs and spleen Tregs was found. We further performed a correlation analysis between miR-21 and Peli1 in autoimmune POI patients and demonstrated that Peli1 had positive correlation with miR-21. Conversely, Marquez et al. [16] observed an inverse correlation between miR-21 and Peli1 mRNA levels during liver regeneration. In the myocardium, Luther et al. [18] found that miR-21a-5p downregulated the expression of proapoptotic gene Peli1. At present, few researches focus on the association between miR-21 and Peli1, especially in autoimmune POI. Thus, the inconsistency of our results may attribute to the various conditions, under which miR-21 might be positively or negatively correlated with Peli1. Moreover, we highlighted the role of Peli1 in immune tolerance, but not in other biological functions such as apoptosis or regeneration. Therefore, the opposite correlation in our study may due to the specific function of Peli1 in different diseases, which consist of separate mechanisms. Generally, it is likely that miR-21 may facilitate the development of autoimmune POI via downregulating Peli1.

Recently, how Peli1 participates in autoimmune POI still remains unknown. Previous studies revealed that the major signaling function of Peli1 in autoimmune disorders was the MAPK or NF-*κ*B pathways. It was reported that Peli1 regulates the MyD88-dependent TLR pathways via ubiquitin-dependent upregulation of cIAP1/2 [31]. Upon activation by the TLR signals, Peli1, as an E3 ligase for cIAP1/2, mediates K63-linked ubiquitination and catalytic activation of cIAP1/2 in microglia. Activated cIAP1/2 can also mediate K48 ubiquitin ligase activity and promote ubiquitin-dependent degradation of TRAF3, leading to MAPK signaling pathway activation [19]. Another study has demonstrated the protective role of Peli1 in SLE pathology by inhibiting noncanonical NF-*κ*B activation, expression of proinflammatory cytokines, and B cell antibody production [21]. In addition, Peli1-deficient mice spontaneously developed SLE through dysregulating the NF-*κ*B family of transcription factors c-Rel. Further studies are needed to be carried out to explore the specific signaling pathway of Peli1 in autoimmune POI condition.

## 6. Conclusions

The present study discovered the low expression of miR-21 and Peli1 in autoimmune POI patients and mouse models. Moreover, in patients, miR-21 had a positive correlation with Peli1, AMH, E_2_, the size of the uterus, and ovarian volume and had a negative correlation with FSH, LH, and the number of positive immune parameters (AOAb, EMAb, ACL, ANA, ds-DNA, ACA, IgG, IgA, IgM, IgE, C3, and C4). Overall, our study revealed the potential mechanism of miR-21 in autoimmune POI, which might involve the ratio of Treg via regulating the target gene Peli1.

## Figures and Tables

**Table 1:** Primers used for qRT-PCR.

Primer	Primer sequence (5′-3′)
Peli1 (forward)	TGGTCCCTATGTCCCTCTGT
Peli1 (reverse)	TGCGTACCATGAGGAAGTG
GAPDH (forward)	GGCCTCCAAGGAGTAAGAAA
GAPDH (reverse)	GCCCCTCCTGTTATTATGG
miR-21 (forward)	ACACTCCAGCTGGGTAGCTTATCAGACTGA
miR-21 (reverse)	CTCAACTGGTGTCGTGGAGTCGGCAATTCAGTTGAGTCAACATC
Cel-mirR-39 mimics (forward)	GACTTCATCACCGGGTGTAAATC
Cel-mirR-39 mimics (reverse)	TATCGTTGTTCTCCACTCCTTGAC

**Table 2:** Hormone levels of mice.

Hormone	POI	Control	*P* value
E_2_ (pg/mL)	30.97 ± 3.51	44.09 ± 4.87	≤0.001
AMH (ng/mL)	4.17 ± 0.52	7.87 ± 0.91	≤0.001
FSH (IU/L)	4.20 ± 0.86	1.94 ± 0.32	0.001

**Table 3:** General characteristics and menstrual condition of patients in both groups.

	POI	Control	*P* value
Age (year)	30.92 ± 7.21	31.43 ± 4.73	0.759
BMI (kg/m^2^)	20.83 ± 2.54	19.82 ± 1.68	0.091
Menopause symptoms	5.00	1.00	≤0.001
Menarche age (year)	14.62 ± 1.33	13.77 ± 0.90	0.016
Menstrual period (day)	3.96 ± 1.641	5.50 ± 1.20	≤0.001
Menstrual cycle (month)	2.54 ± 1.17	1.00 ± 0.00	≤0.001
Gravidity	0	2.000	≤0.001
Parity	0	1.000	≤0.001

**Table 4:** Hormone levels of POI patients and normal cycling women.

Hormone	POI	Control	*P* value
FSH (IU/L)	78.27 ± 40.39	7.57 ± 2.72	≤0.001
LH (IU/L)	38.78 ± 22.31	6.12 ± 6.42	≤0.001
E_2_ (pmol/L)	117.60 ± 131.15	268.70 ± 279.03	≤0.001
AMH (*μ*g/L)	0.05 ± 0.08	1.72 ± 0.28	≤0.001
fT3 (*ρ*mol/L)	4.97 ± 1.83	5.06 ± 0.51	0.808
fT4 (*ρ*mol/L)	11.60 ± 2.79	11.68 ± 1.22	0.889
TSH (mIU/L)	1.65 ± 1.05	1.56 ± 0.73	0.741

**Table 5:** Spearman's correlation coefficients for the Peli1 of the patients.

	Peli1	
	*r*	*P* value
miR-21	0.719	≤0.001
Number of positive immune parameters	-0.842	≤0.001
E_2_	0.460	≤0.001
FSH	-0.687	≤0.001
LH	-0.706	≤0.001
AMH	0.706	≤0.001
Size of the uterus	0.439	0.001
Ovarian volume	0.581	≤0.001
Endometrial thickness	0.257	0.056

**Table 6:** Spearman's correlation coefficients for the miR-21 of the patients.

	miR-21	
	*r*	*P* value
Number of positive immune parameters	-0.850	≤0.001
E_2_	0.436	0.001
FSH	-0.773	≤0.001
LH	-0.684	≤0.001
AMH	0.765	≤0.001
Size of the uterus	0.460	≤0.001
Ovarian volume	0.649	≤0.001
Endometrial thickness	0.335	0.012

## Data Availability

The data used to support the findings of this study are available from the corresponding author upon request.
